# Diagnostic utility of CSF1 immunohistochemistry in tenosynovial giant cell tumor for differentiating from giant cell-rich tumors and tumor-like lesions of bone and soft tissue

**DOI:** 10.1186/s13000-022-01266-9

**Published:** 2022-11-01

**Authors:** Shintaro Sugita, Tomoko Takenami, Tomomi Kido, Tomoyuki Aoyama, Michiko Hosaka, Keiko Segawa, Taro Sugawara, Hiromi Fujita, Junya Shimizu, Yasutaka Murahashi, Makoto Emori, Tadashi Hasegawa

**Affiliations:** 1 Department of Surgical Pathology, School of Medicine, Sapporo Medical University, 060-8543 Sapporo, Hokkaido Japan; 2 Department of Orthopedic Surgery, School of Medicine, Sapporo Medical University, 060-8543 Sapporo, Hokkaido Japan

**Keywords:** Tenosynovial giant cell tumor, Localized type, Diffuse type, CSF1, Immunohistochemistry, Fluorescence in situ hybridization

## Abstract

**Background:**

Tenosynovial giant cell tumor (TSGCT) is a benign fibrohistiocytic tumor that affects the synovium of joints, bursa, and tendon sheaths and is categorized into localized TSGCT (LTSGCT) and diffuse TSGCT (DTSGCT). LTSGCT and DTSGCT are characterized by recurrent fusions involving the colony-stimulating factor 1 (*CSF1*) gene and its translocation partner collagen type VI alpha 3 chain. The fusion gene induces intratumoral overexpression of *CSF1* mRNA and CSF1 protein. CSF1 expression is a characteristic finding of TSGCT and detection of *CSF1* mRNA and CSF1 protein may be useful for the pathological diagnosis. Although there have been no effective anti-CSF1 antibodies to date, in situ hybridization (ISH) for *CSF1* mRNA has been performed to detect *CSF1* expression in TSGCT. We performed CSF1 immunohistochemistry (IHC) using anti-CSF1 antibody (clone 2D10) in cases of TSGCT, giant cell-rich tumor (GCRT), and GCRT-like lesion and verified its utility for the pathological diagnosis of TSGCT.

**Methods:**

We performed CSF1 IHC in 110 cases including 44 LTSGCTs, 20 DTSGCTs, 1 malignant TSGCT (MTSGCT), 10 giant cell tumors of bone, 2 giant cell reparative granulomas, 3 aneurysmal bone cysts, 10 undifferentiated pleomorphic sarcomas, 10 leiomyosarcomas, and 10 myxofibrosarcomas. We performed fluorescence ISH (FISH) for *CSF1* rearrangement to confirm CSF1 expression on IHC in TSGCTs. We considered the specimens to have *CSF1* rearrangement if a split signal was observed in greater than 2% of the tumor cells.

**Results:**

Overall, 50 of 65 TSGCT cases, including 35 of the 44 LTSGCTs and 15 of the 20 DTSGCTs, showed distinct scattered expression of CSF1 in the majority of mononuclear tumor cells. MTSGCT showed no CSF1 expression. Non-TSGCT cases were negative for CSF1. FISH revealed *CSF1* rearrangement in 6 of 7 CSF1-positive cases on IHC. On the other hand, FISH detected no *CSF1* rearrangement in all CSF1-negative cases on IHC. Thus, the results of IHC corresponded to those of FISH.

**Conclusion:**

We revealed characteristic CSF1 expression on IHC in cases of TSGCT, whereas the cases of non-TSGCT exhibited no CSF1 expression. CSF1 IHC may be useful for differentiating TSGCTs from histologically mimicking GCRTs and GCRT-like lesions.

## Introduction

The current World Health Organization classification of soft tissue and bone tumors defines tenosynovial giant cell tumor (TSGCT) as a benign fibrohistiocytic tumor that affects the synovium of joints, bursa, and tendon sheaths [1]. TSGCT is divided into two main types: localized TSGCT (LTSGCT) and diffuse TSGCT (DTSGCT). In addition to these, malignant TSGCT (MTSGCT) has rarely been reported. LTSGCT arises predominantly in the digits of the hand and foot. DTSGCT commonly affects the intra-auricular region of the large joints including knee, hip, ankle, elbow, and shoulder joints. On the other hand, MTSGCTs are highly aggressive sarcomas with metastases to the lymph nodes and lung and have a poor prognosis [2, 3].

Histologically, LTSGCT shows a well-demarcated lobulated mass that consists of various proportions of mononuclear cells, osteoclast-like giant cells, foamy cells, and inflammatory infiltrates. The stroma shows hemosiderin deposits and various degrees of hyalinization. By contrast, DTSGCT usually exhibits an infiltrative growth pattern with diffuse and expansile sheets of various tumor components and commonly shows a cleft-like space with severe hemosiderin deposits. Therefore, LTSGCT and DTSGCT differ in clinical presentation and histology, although the pathogenesis of these tumors is characterized by fusion of the colony-stimulating factor 1 (*CSF1*) and collagen type VI alpha 3 chain *(COL6A3)* genes derived from translocation of t(1p13; 2q35) [4]. The fusion gene induces overexpression of *CSF1* mRNA and CSF1 protein (also called macrophage colony stimulation factor) in the tumor. Therefore, CSF1 expression is a characteristic finding of TSGCT, and CSF1 expression leads to the recruitment of macrophages to the tumor tissue of osteoclast-like giant cells. The characteristic histology of TSGCT is the intermingling of mononuclear cells with many inflammatory cells and osteoclast-like giant cells [4, 5]. Because CSF1 expression is characteristic in TSGCT, detecting *CSF1* mRNA and CSF1 protein may be useful for the pathological diagnosis of TSGCT. Although there are no effective anti-CSF1 antibodies to date, *CSF1* mRNA in situ hybridization (ISH) has been performed to detect CSF1 expression in TSGCT [5, 6].

In this study, we performed CSF1 immunohistochemistry (IHC) using anti-CSF1 antibody (clone 2D10) in the pathological specimens of TSGCT patients and verified its utility for the pathological diagnosis of TSGCT. In addition, we evaluated *CSF1* rearrangement in TSGCT by fluorescence ISH (FISH) using a dual-color break-apart probe. We also investigated CSF1 IHC in giant cell-rich tumor (GCRT) and GCRT-like lesion, which occur in the bone and characteristically show the presence of osteoclast-like giant cell including giant cell tumor of bone, giant cell reparative granuloma, and aneurysmal bone cyst. Moreover, we performed CSF1 IHC in undifferentiated pleomorphic sarcoma, leiomyosarcoma, and myxofibrosarcoma (MFS) as these tumors may contain osteoclast-like giant cells.

## Materials and methods

### Sample selection

This study was performed with approval of the Institutional Review Board (IRB) of Sapporo Medical University Hospital (No. 342 − 33). We selected a total of 110 cases from our pathological archives including 44 LTSGCTs, 20 DTSGCTs, 1 MTSGCT, 10 giant cell tumors of bone, 2 giant cell reparative granulomas, 3 aneurysmal bone cysts, 10 undifferentiated pleomorphic sarcomas, 10 leiomyosarcomas, and 10 myxofibrosarcomas. We performed hematoxylin and eosin (H&E) staining using 3-µm-thick sections. We reviewed all H&E-stained slides and previously stained IHC slides and confirmed the pathological diagnosis in individual cases.

## Immunohistochemistry

We performed IHC for CSF1 using representative sections from formalin-fixed and paraffin-embedded tissues from individual cases. These tissues were sliced into 3-µm-thick sections and examined with an automated IHC system at Sapporo Medical University Hospital. All slides were loaded into a PT Link Module (Agilent Technologies, Santa Clara, CA) and subjected to a heat-induced antigen-retrieval protocol with the EnVision FLEX Target Retrieval Solution (Agilent Technologies) before being transferred to Autostainer Link 48 (Agilent Technologies). We used commercially available antibodies against CSF1 (clone 2D10, 1:500 dilution; Millipore Corporation, Temecula, CA). CSF1 expression was considered positive if foci of scattered CSF1-positive cells with discernible cytoplasmic expression were diffusely or at least focally observed.

### Fluorescence in situ hybridization

We performed FISH for *CSF1* rearrangement to confirm CSF1 expression detected by IHC in 7 CSF1-positive and 5 CSF1-negative cases. We also performed FISH for MTGCT. We used the commercially available *CSF1* dual-color break-apart probe (CSF1 Split Dual Color FISH Probe; GSP Lab., Inc., Hyogo, Japan). FISH was performed as previously described [7]. Briefly, the specimens were tumor tissues in 4-µm-thick slices on glass slides. We first selected an area showing representative histology and marked a 5-mm circle with a marker pen on the glass slide. We used the PathVysion HER-2 DNA Probe Kit (Abbott Laboratories, Chicago, IL) according to the manufacturer’s instructions with the following modifications: baking (60 °C for 1 h), deparaffinization, target gene activation (20 min with 0.2 M HCl followed by 80 °C for 30 min with pretreatment solution), enzyme treatment (37 °C for 60 min with protease solution), re-fixation (10 min with 10% formalin neutral buffer solution), denaturation (72 °C for 5 min with denaturation solution), washing and dehydration (1 min each in 70%, 85%, and 100% ethanol), hybridization with 10 mL DNA probe solution (90 °C for 5 min, followed by 37 °C for 48 h), and washing with post-hybridization wash buffer (72 °C for 2 min). For counterstaining, 10 µL of 4,6-diamidino-2-phenylindole was added. The slides were coverslipped for viewing under a fluorescence microscope. We counted 50 nuclei and defined the signals as split when the distance between the red and green signals was at least twice the estimated signal diameter. We considered the specimens to have *CSF1* rearrangement if a split signal was observed in greater than 2% of the tumor cells　[6].

## Results

### Clinical features

The clinical findings of the 110 cases examined are summarized in Table 1. Patients with LTSGCT included 21 males and 23 females (mean age 47, range 9–78 years). A digit of the hand or foot was the most affected anatomical site (n = 32). Patients with DTSGCT included 5 males and 15 females (mean age 36, range 17–58 years). The knee was the most affected anatomical site (n = 12). Rare anatomical sites included vertebra (n = 1). The patient with MTSGCT was a 22-year-old man who had an affected digit. He underwent the local recurrence 3 times and the last recurrent tumor showed malignant transformation. Details of clinical features of the non-TSGCT cases were also demonstrated in Table 1.

**Table 1:** Clinical findings of the 110 cases examined

Histological type	Mean age (years)	Sex	Site
Localized tenosynovial giant cell tumor (44)	47 (9–78)	M (21)	Digit (32)
		F (23)	Hand (6)
			Foot (5)
			Knee (1)
Diffuse tenosynovial giant cell tumor (20)	36 (17–58)	M (5)	Knee (12)
		F (15)	Hip (2)
			Foot (2)
			Shoulder (1)
			Elbow (1)
			Digit (1)
			Vertebra (1)
Malignant tenosynovial giant cell tumor (1)	22	M (1)	Digit (1)
Giant cell tumor of bone (10)	43 (26–60)	M (1)	Femur (5)
		F (9)	Tibia (3)
			Humerus (1)
			Radius (1)
Aneurysmal bone cyst (3)	15 (13–16)	M (1)	Fibula (1)
		F (2)	Ilium (1)
			Sacrum (1)
Giant cell reparative granuloma (2)	53 (31–75)	M (1)	Cranium (1)
		F (1)	Jaw (1)
Undifferentiated pleomorphic sarcoma (10)	71 (62–87)	M (5)	Lower extremity (5)
		F(5)	Trunk (3)
			Upper extremity (1)
			Shoulder (1)
Leiomyosarcoma (10)	81 (58–92)	M (4)	Lower extremity (6)
		F (6)	Upper extremity (2)
			Trunk (2)
Myxofibrsarcoma (10)	70 (47–88)	M (7)	Upper extremity (6)
		F (3)	Lower extremity (4)

M, male; F, female

## Histological findings

**Fig. 1:**
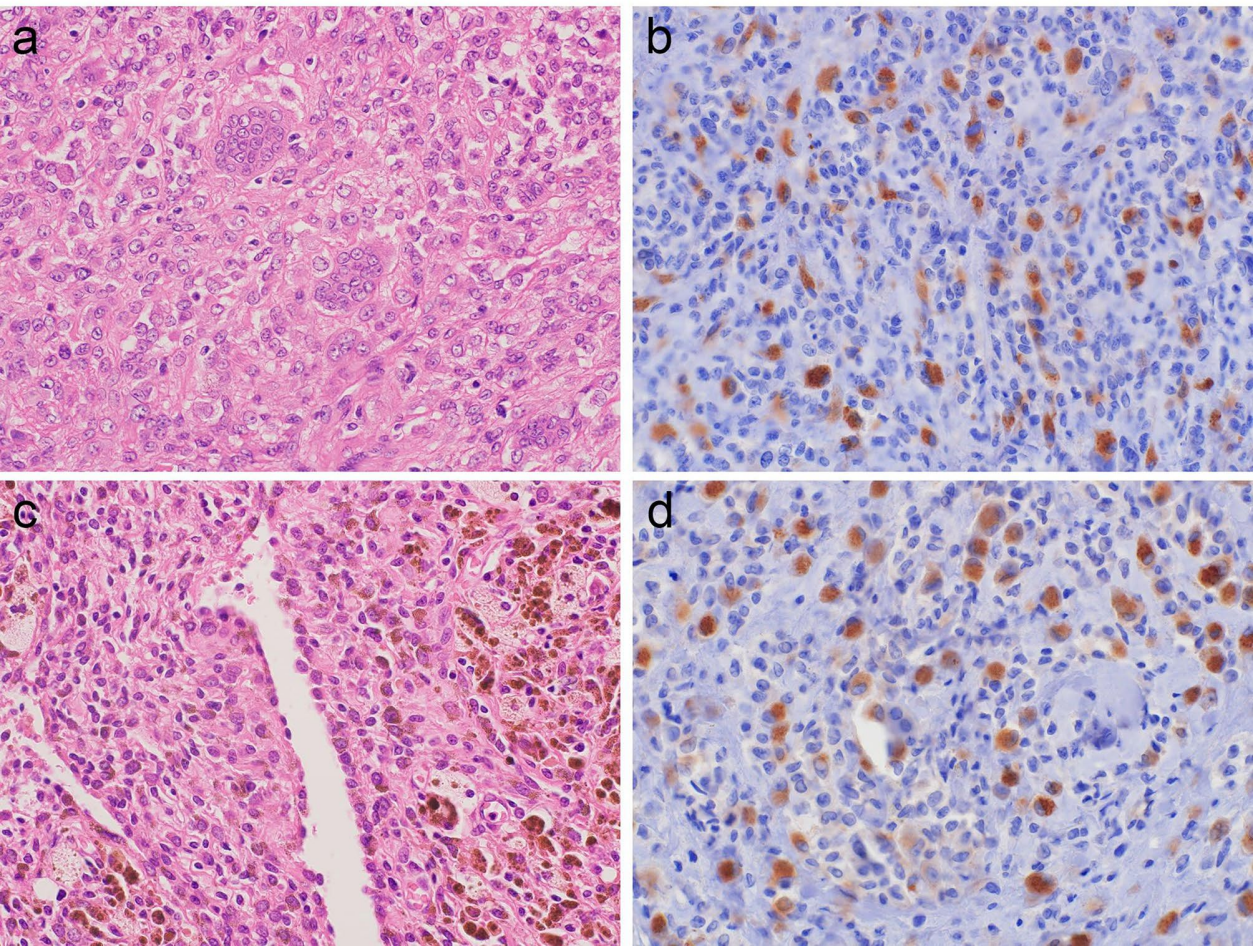
**Histology and CSF1 IHC of TSGCTs.** a. LTSGCT showed a well-circumscribed mass that consisted of admixture of mononuclear cells, osteoclast-like giant cells, and inflammatory infiltrates with various degrees of collagenous stroma b. CSF1-positive mononuclear tumor cells were scattered in the tumor c. DTSGCT was composed of various cells similar to LTSGCT that exhibited sheet proliferation with often cleft-like structures and marked hemosiderin deposits d. CSF1-positive mononuclear tumor cells were scattered in the tumor

**Fig. 2:**
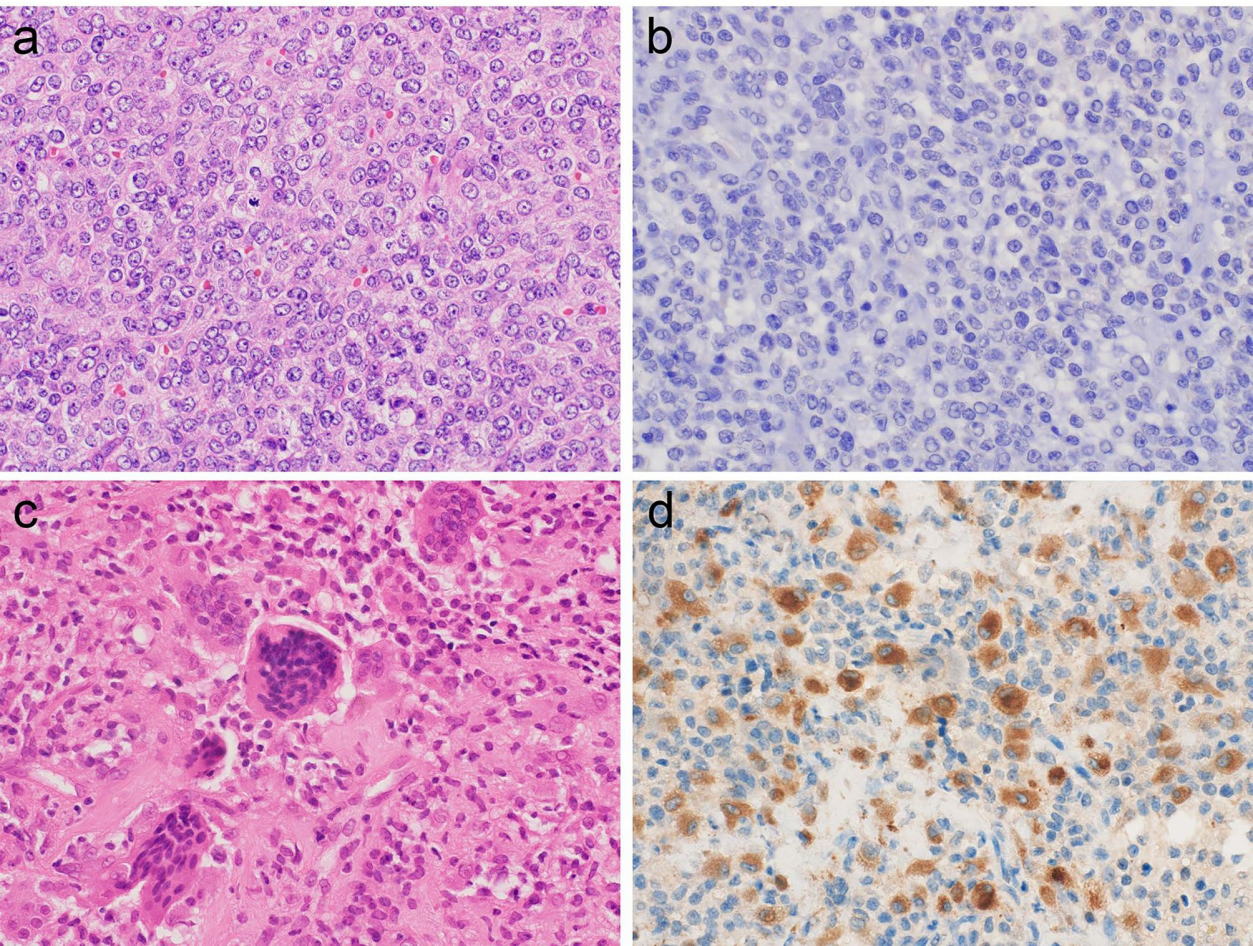
**Pathological findings of MTSGCT and DTSGCT originated in the intervertebral joint of thoracic vertebra** a. MTSGCT consisted of diffuse proliferation of round to ovoid mononuclear tumor cells with coarse chromatin and conspicuous nucleoli. Mitotic figures were observed b. The mononuclear tumor cells were negative for CSF1. c. DTSGCT arose in the intervertebral joint of thoracic vertebra that consists of admixture of mononuclear cells, osteoclast-like giant cells, and inflammatory infiltrates with various degrees of collagenous stroma d. CSF1-positive mononuclear tumor cells were scattered in the tumor. This case was initially diagnosed as giant cell reparative granuloma but finally determined to be DTSGCT involving the vertebra by CSF1 IHC.

**Fig. 3:**
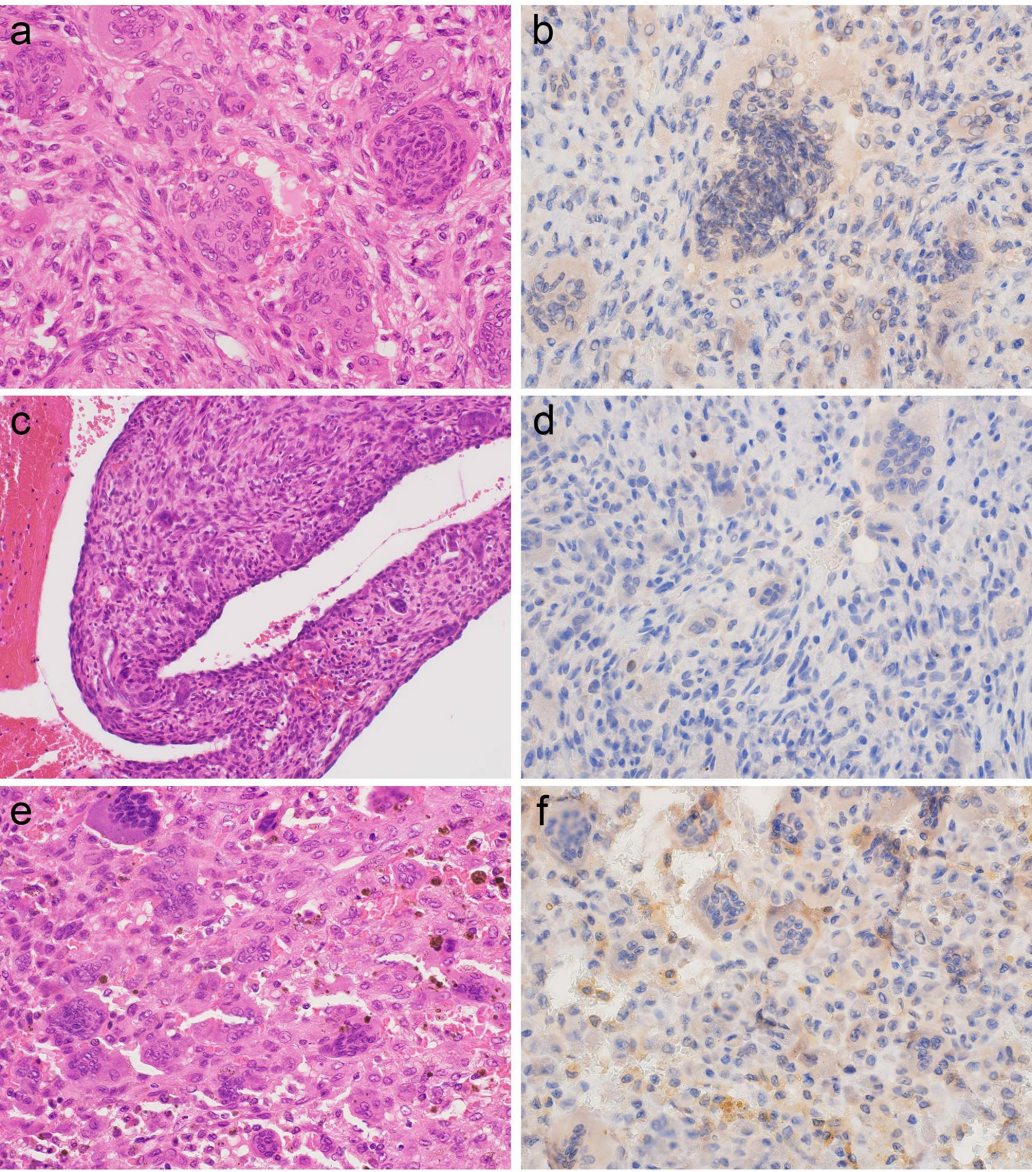
**Histology and CSF1 immunohistochemistry of giant cell tumor of bone, aneurysmal bone cyst, and giant cell reparative granuloma** a. Giant cell tumor of bone consisted of solid proliferation of histiocyte-like mononuclear cells with many osteoclast-like giant cells b. The mononuclear tumor cells were negative for CSF1. c. Aneurysmal bone cyst exhibited a blood-filled cystic space separated by fibrous septa. The fibrous septa consisted of mononuclear and fibroblastic spindle cells with osteoclast-like giant cells d. The mononuclear cells were negative for CSF1. e. Giant cell reparative granuloma was composed of diffuse proliferation of mononuclear to spindle cells without atypia accompanying osteoclast-like giant cells f. The mononuclear cells were negative for CSF1.

**Fig. 4:**
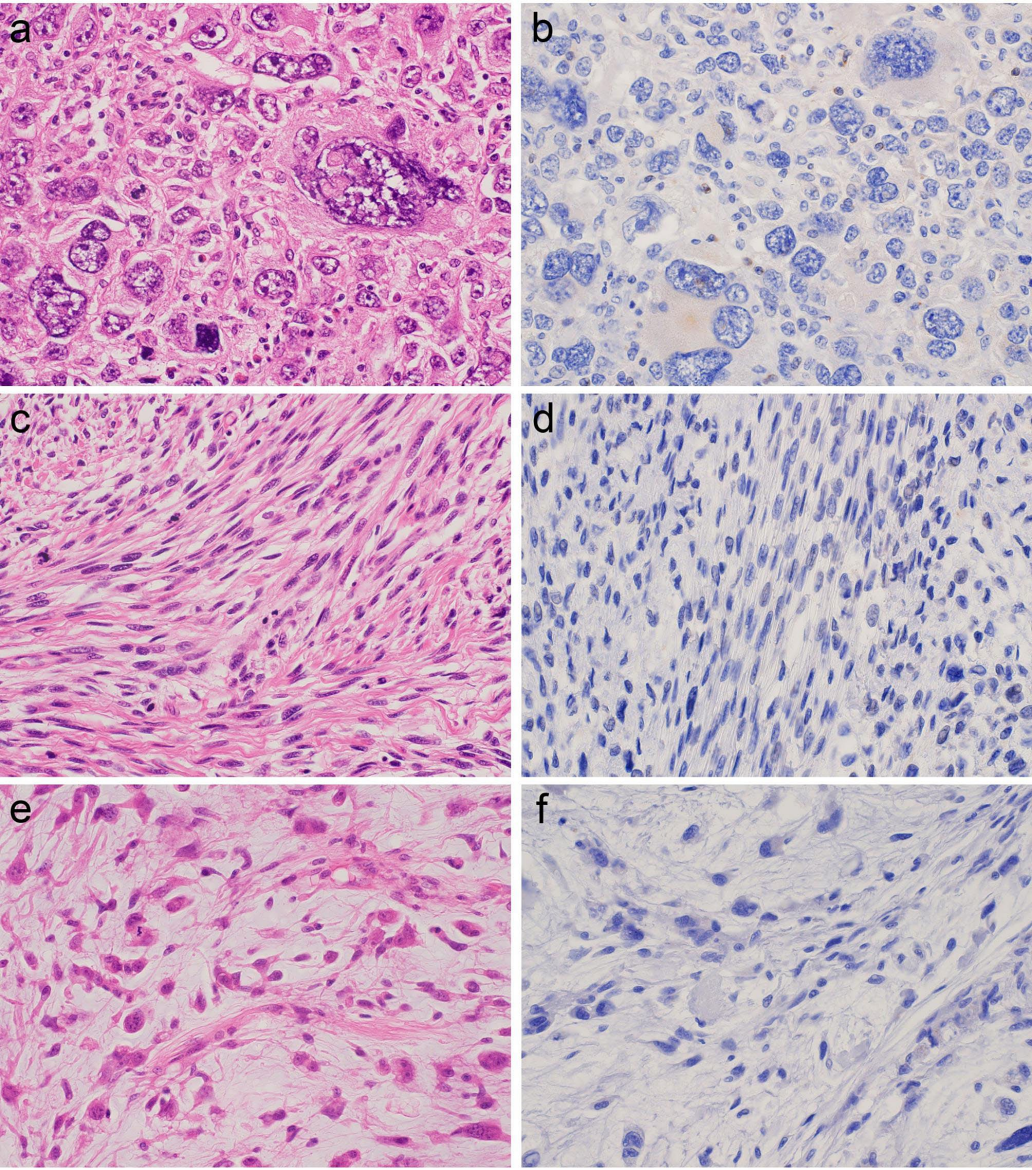
**Histology and CSF1 IHC of undifferentiated pleomorphic sarcoma, leiomyosarcoma, and myxofibrosarcoma** a. Undifferentiated pleomorphic sarcoma showed a solid proliferation of atypical spindle to pleomorphic cells with marked nuclear atypia and nuclear pleomorphism b. The tumor cells were negative for CSF1. c. Leiomyosarcoma was composed of a fascicular proliferation of eosinophilic spindle cells with cigar-like elongated nuclei d. The tumor cells were negative for CSF1. e. Myxofibrosarcoma was composed of a fascicular proliferation of atypical spindle cells with vesicular nuclei with moderate to severe nuclear pleomorphism. The background was myxoid f. The tumor cells were negative for CSF1.

Histologically, LTSGCT showed a well-circumscribed mass consisting of admixture of mononuclear cells, osteoclast-like giant cells, foamy cells, and inflammatory infiltrates with various degrees of collagenous stroma (Fig. 1a). DTSGCT was also composed of various cells similar to LTSGCT that exhibited sheet proliferation with often cleft-like structures and marked hemosiderin deposits (Figs. 1c and 2c). MTSGCT consisted of diffuse proliferation of round to ovoid mononuclear tumor cells with coarse chromatin and conspicuous nucleoli (Fig. 2a). Mitotic figures were frequently observed. The initial and secondary recurrent tumors showed the histology of conventional TSGCT. The morphologic features of the non-TSGCT cases were demonstrated in Figs. 3 and 4.

**Fig. 5:**
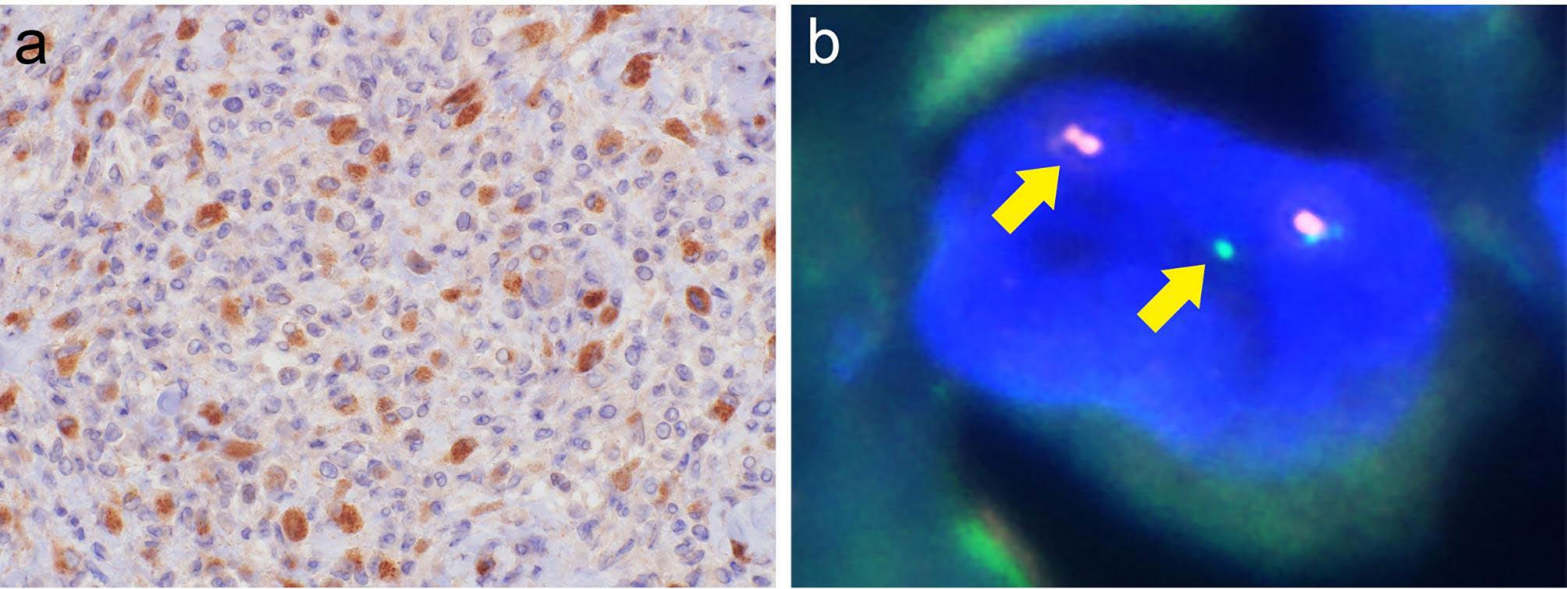
**CSF1 IHC and** ***CSF1*** **split signal by FISH in TSGCT.** a. CSF1-positive mononuclear tumor cells were scattered in the tumor b. The tumor cells showed a split signal pattern that consisted of a pair of split (red and green) and fused signals. The split signal was observed in 10% of tumor cells

## Immunohistochemistry

The results of IHC are summarized in Table 2. The majority of TSGCT cases were positive for CSF1 on IHC. A total of 50 of 65 (77%) TSGCTs, including 35 of 44 (79.5%) LTSGCTs (Fig. 1b) and 15 of 20 (75%) DTSGCTs (Figs. 1d and 2d), were positive for CSF1. In the CSF1-positive cases, many CSF1-positive cells were scattered in the tumor and discernable cytoplasmic staining was seen regardless of its intensity. One case of MTSGCT showed no expression of CSF1 (Fig. 2b) although the initial and secondary recurrent tumors showed weak CSF1 expression. On the other hand, no cases of giant cell tumor of bone (Fig. 3b), aneurysmal bone cyst (Fig. 3d), or giant cell reparative granuloma (Fig. 3f) exhibited CSF1 expression. All cases of undifferentiated pleomorphic sarcoma (Fig. 4b), leiomyosarcoma (Fig. 4d), and myxofibrosarcoma (Fig. 4f) were negative for CSF1.

**Table 2:** CSF1 expression by IHC in giant cell-rich tumors and tumor-like lesions of the bone and soft tissue

Histological type	Total (n)	CSF1-positive (n)	Percent (%)
Tenosynovial giant cell tumor	65	50	77
Localized type	44	35	79.5
Diffuse type	20	15	75.0
Malignant	1	0	0
Giant cell tumor of bone	10	0	0
Aneurysmal bone cyst	3	0	0
Giant cell reparative granuloma	2	0	0
Undifferentiated pleomorphic sarcoma	10	0	0
Leiomyosarcoma	10	0	0
Myxofibrosarcoma	10	0	0

### Fluorescence in situ hybridization

FISH revealed *CSF1* split signals in 0%, 4%, 6%, 6%, 10%, 10%, and 26% of tumor cells in 7 TSGCTs (4 LTSGCTs and 3 DTSGCTs) that were positive for CSF1 on IHC (Fig. 5a, b). In the CSF1-positive cases on IHC, 6 of 7 (86%) were positive for *CSF1* rearrangement by FISH. On the other hand, FISH detected *CSF1* split signals in 0%, 0%, 2%, 2%, and 2% in 5 TSGCTs (4 LTSGCTs and 1 DTSGCT) that were negative for CSF1 on IHC. In the CSF1-negative cases on IHC, no case showed CSF1 rearrangement by FISH. Thus, the results of IHC corresponded to those of FISH. MTSGCT showed *CSF1* split signals in 2% of tumor cells and so no *CSF1* rearrangement.

## Discussion

Because GCRTs including TSGCTs and GCRT-like lesions arising in the bone and soft tissue often exhibit similar morphology that consists of sheet-like proliferation of histiocytic mononuclear cells with characteristic osteoblast-like giant cells, inflammatory cells, and aggregation of foam cells, their differential diagnosis requires clinical information including age, sex, location, and radiological findings in addition to histological examination. Therefore, a precise diagnosis may be difficult if clinical and radiological findings are not typical. In this study, we found specific CSF1 expression in both LTSGCT and DTSGCT cases. Conversely, no CSF1 expression was observed in any case of giant cell tumor of bone, aneurysmal bone cyst, or giant cell reparative granuloma. Therefore, we conclude that CSF1 IHC is a useful diagnostic tool for the differential diagnosis of TSGCTs. We experienced a challenging case of vertebral TSGCT as illustrated in Fig. 2c and d. Our first impression of the case was aneurysmal bone cyst although FISH detected no *USP6* split signal in the tumor cells. Therefore, we initially diagnosed the case as GCRT-like lesion with suspected giant cell reparative granuloma. However, as this case showed distinct CSF1 expression on IHC, we finally diagnosed the patient with DTSGCT arising from the intervertebral joint of the spine involving the vertebral bone combined with careful examination of the radiological findings. Thus, CSF1 IHC is effective for the confirmative diagnosis of TSGCT if the location of the tumor is unusual.

CSF1 is a homodimeric glycoprotein that is required for the lineage-specific growth of cells of the mononuclear phagocyte series. CSF1 primarily regulates the survival, proliferation, and differentiation of monocytes/macrophages, which sustains the protumorigenic functions of tumor-associated macrophages [8, 9]. In TSGCTs, the tumor cells have *CSF1* rearrangement and it induces CSF1 overexpression, which leads to the mobilization of many inflammatory cells and osteoclast-like giant cells into the tumor tissue [4, 5]. CSF1 expression has been found in musculoskeletal tumors including TSGCT and leiomyosarcoma by ISH or IHC [5, 6, 10, 11], although no study has focused on the diagnostic utility of CSF1 IHC for the differential diagnosis between TSGCTs and giant cell-rich lesions including giant cell tumor of bone, aneurysmal bone cyst, and giant cell reparative granuloma.

Some previous studies have examined *CSF1* mRNA expression by ISH and chromogenic ISH (CISH) in cases of TSGCT and non-tumoral synovium [5, 6, 10]. They revealed characteristic *CSF1* mRNA expression in almost all TSGCT cases by ISH (96%) and CISH (100%), respectively [5, 10]. *CSF1* mRNA expression showed a scattered pattern throughout the tumors as shown in our CSF1 IHC study. CSF1 expression using IHC has scarcely been examined in the musculoskeletal tumors due to the lack of reliable CSF1 antibodies for formalin-fixed and paraffin-embedded Sect. [6]. In a study of CSF1 IHC tested in TSGCT cases by Cupp et al. [5], CSF1 expression was present diffusely throughout the lesions of mononuclear cells in 42 of 51 cases (82%). However, interpretation of the intensity of localization was difficult because of the high background of staining present. On the other hand, they examined CSF1 expression in reactive synovitis and CSF1 staining showed a linear pattern that highlighted the synovial lining. Moreover, they analyzed *CSF1* mRNA ISH in various types of soft tissue tumor including leiomyosarcoma and undifferentiated pleomorphic sarcoma and *CSF1* mRNA expression pattern was similar to the punctate pattern seen in TSGCTs. Our study showed that CSF1 expression was present in 50 of 65 (77%) TSGCT cases by IHC in which many CSF1-positive cells were scattered in the tumor and discernable cytoplasmic staining was seen regardless of its intensity. We found CSF1 expression only in cases of TSGCTs and not in GCRTs or GCRT-like lesions. Taken together, our results suggest that CSF1 IHC is an effective diagnostic tool for TSGCTs.

In our study, a case of MTSGCT exhibited no CSF1 expression on IHC. A previous study revealed non-CSF1 fusions in cases of atypical TSGCT with increased cellular atypia and mitotic activity. These atypical TSGCTs harbored unique non-CSF1 gene fusions including *NIPBL-ERG*, *FN1-ROS1*, and *YAP1-MAML2* [12]. Although we rarely detected *CSF1* split signals in the tumor cells of MTGCT, it is possible that these or new fusion genes may exist in these cases.

Recently, Agaimy et al. have reported distinctive giant cell-rich soft tissue neoplasm that expressed keratins and carried a recurrent *HMGA2-NCOR2* gene fusion [13]. Histologically, the tumor consisted of bland plump epithelioid or ovoid to spindled mononuclear cells mixed with evenly distributed multinucleated osteoclast-type giant cells. On IHC, the mononuclear cells were characteristically positive for cytokeratin AE1/AE3 cocktail. Interestingly, the tumor had a novel *HMGA2-NCOR2* fusing gene and was considered to be a genetically distinct entity of giant cell tumor of soft tissue. This unique tumor of the new entity also should be differentiated from TSGCT and its histological mimics. Differential diagnosis of keratin positive giant cell-rich soft tissue neoplasm from TSGCT may be easy because the former tumor showed the characteristic keratin expression. On the other hand, TSGCT usually exhibited no obvious keratin expression.

A limitation of this study is that TSGCTs generally consist of a mixture of mononuclear tumor cells and various types of non-tumor cells including inflammatory cells, foam cells, and fibroblasts, so we often had difficulty counting signal-positive cells under a fluorescence microscope on FISH even if comparing the morphology of H&E-stained slides. Thus, we may have underestimated the positive rate of cells with split signals because of the similarity between mononuclear tumor cells and inflammatory cells. Mastboom et al. [6] reported a cutoff value of *CSF1* split signal of greater than 2% in their FISH analysis. Adapting this cutoff value, they detected *CSF1* rearrangement in 76% of all TSGCT cases including 77% in LTSGCT and 75% in DTSGCT cases. However, this cutoff value is apparently lower than that of general split FISH in translocation-related sarcomas (10% or 20%) [7]. For example, we usually adapt 10% as a cut-off value of split signals in tumors consisting of cytologically monomorphic cells such as Ewing sarcoma and synovial sarcoma [14]. In the present study, cases positive for CSF1 on IHC showed *CSF1* split signals in greater than 2% of tumor cells. By contrast, cases negative for CSF1 on IHC exhibited no or only 2% *CSF1* split signal of tumor cells. Therefore, we should carefully interpret the *CSF1* split signal as “positive” on FISH combined with the results of CSF1 IHC.

## Conclusion

We revealed characteristic CSF1 expression on IHC in only cases of TSGCT and not in GCRTs and GCRT-like lesions such as giant cell tumor of bone, aneurysmal bone cyst, giant cell reparative granuloma, undifferentiated pleomorphic sarcoma, leiomyosarcoma, and myxofibrosarcoma. The distinctive CSF1 expression in TSGCTs was scattered in many tumor cells. We conclude that CSF1 IHC may be a useful diagnostic tool to differentiate TSGCTs from histologically mimicking GCRTs and GCRT-like lesions of bone and soft tissues.

## Data Availability

Not applicable.

## Abbreviations

TSGCT: tenosynovial giant cell tumor
IHC: immunohistochemistry
FISH: fluorescence in situ hybridization

## References

[1] von de Rijn M, Antonescu CR, Blay JV, Bovee JVMG, de Saint Aubain Somerhausen N (2020). Tenosynovial giant cell tumor, WHO classification of tumours of soft tissue and bone.

[2] Nakayama R, Jagannathan JP, Ramaiya N, Ferrone ML, Raut CP, Ready JE (2018). Clinical characteristics and treatment outcomes in six cases of malignant tenosynovial giant cell tumor: initial experience of molecularly targeted therapy. BMC Cancer.

[3] Al-Ibraheemi A, Ahrens WA, Fritchie K, Dong J, Oliveira AM, Balzer B (2019). Malignant Tenosynovial Giant Cell Tumor: The True “Synovial Sarcoma?“ A Clinicopathologic, Immunohistochemical, and Molecular Cytogenetic Study of 10 Cases, Supporting Origin from Synoviocytes. Mod Pathol.

[4] West RB, Rubin BP, Miller MA, Subramanian S, Kaygusuz G, Montgomery K (2006). A landscape effect in tenosynovial giant-cell tumor from activation of CSF1 expression by a translocation in a minority of tumor cells. Proc Natl Acad Sci U S A.

[5] Cupp JS, Miller MA, Montgomery KD, Nielsen TO, O’Connell JX, Huntsman D (2007). Translocation and expression of CSF1 in pigmented villonodular synovitis, tenosynovial giant cell tumor, rheumatoid arthritis and other reactive synovitis. Am J Surg Pathol.

[6] Mastboom MJL, Hoek DM, Bovée JVMG, van de Sande MAJ, Szuhai K (2019). Does CSF1 overexpression or rearrangement influence biological behaviour in tenosynovial giant cell tumours of the knee?. Histopathology.

[7] Sugita S, Asanuma H, Hasegawa T (2016). Diagnostic use of fluorescence in situ hybridization in expert review in a phase 2 study of trabectedin monotherapy in patients with advanced, translocation-related sarcoma. Diagn Pathol.

[8] Sherr CJ, Roussel MF, Rettenmier CW (1988). Colony-stimulating factor-1 receptor (c-fms). J Cell Biochem.

[9] Fujiwara T, Yakoub MA, Chandler A, Christ AB, Yang G, Ouerfelli O (2021). CSF1/CSF1R Signaling Inhibitor Pexidartinib (PLX3397) Reprograms Tumor-Associated Macrophages and Stimulates T-cell Infiltration in the Sarcoma Microenvironment. Mol Cancer Ther.

[10] Thangaiah JJ, Koepplin JW, Folpe AL (2021). RNAscope CSF1 chromogenic in situ hybridization: a potentially useful tool in the differential diagnosis of tenosynovial giant cell tumors. Hum Pathol.

[11] Espinosa I, Edris B, Lee CH, Cheng HW, Gilks CB, Wang Y (2011). CSF1 expression in nongynecological leiomyosarcoma is associated with increased tumor angiogenesis. Am J Pathol.

[12] Vougiouklakis T, Shen G, Feng X, Hoda ST, Jour G (2019). Molecular Profiling of Atypical Tenosynovial Giant Cell Tumors Reveals Novel Non-CSF1 Fusions. Cancers (Basel).

[13] Agaimy A, Michal M, Stoehr R, Ferrazzi F, Fabian P, Michal M (2021). Recurrent novel *HMGA2-NCOR2* fusions characterize a subset of keratin-positive giant cell-rich soft tissue tumors. Mod Pathol.

[14] Sugita S, Aoyama T, Ito Y, Asanuma H, Sugawara T, Segawa K (2017). Diagnostic utility of automated SureFISH (Dako Omnis) in the diagnosis of musculoskeletal translocation-related sarcomas. Pathol Int.

